# A New Crustin Gene Homolog SpCrus8 Identified in *Scylla paramamosain* Exerting *In Vivo* Protection Through Opsonization and Immunomodulation

**DOI:** 10.3389/fimmu.2022.946227

**Published:** 2022-07-08

**Authors:** Manyu Jiang, Roushi Chen, Fangyi Chen, Xuewu Zhu, Ke-Jian Wang

**Affiliations:** ^1^State Key Laboratory of Marine Environmental Science, College of Ocean & Earth Sciences, Xiamen University, Xiamen, China; ^2^State-Province Joint Engineering Laboratory of Marine Bioproducts and Technology, College of Ocean & Earth Sciences, Xiamen University, Xiamen, China; ^3^Fujian Innovation Research Institute for Marine Biological Antimicrobial Peptide Industrial Technology, College of Ocean & Earth Sciences, Xiamen University, Xiamen, China

**Keywords:** antimicrobial peptides (AMPs), crustin, phagocytosis, opsonization, immunomodulation

## Abstract

Crustins are the most abundant class of antimicrobial peptides in crustaceans and are essential for protecting animals from infection. Among them, type II crustins usually exhibit potent antimicrobial activity. Interestingly, in this study, a newly identified type II crustin gene homolog (named SpCrus8) from mud crab *Scylla paramamosain*, the recombinant proteins of which (rSpCrus8 and rTrx-SpCrus8) showed no obvious antibacterial effects, but could significantly reduce the bacterial load in crab hemolymph and improve the survival rate of crabs infected with *Vibrio alginolyticus*. The immune-related function of SpCrus8 and the underlying mechanism deserve further investigation. It was found that the SpCrus8 gene was widely distributed in various tissues of adult crabs. In the hepatopancreas of crabs infected with *V. alginolyticus* or *Staphylococcus aureus*, transcripts of the SpCrus8 gene were remarkably induced, indicating that the SpCrus8 gene was involved in the immune response to bacterial infection *in vivo*. In addition, rSpCrus8 and rTrx-SpCrus8 had strong binding activity not only to microbial surface components (lipopolysaccharide, lipoteichoic acid, peptidoglycan, and glucan), but also to the tested bacteria (*S. aureus*, *Pseudomonas aeruginosa* and *V. alginolyticus*). Notably, rSpCrus8 and rTrx-SpCrus8 could significantly promote hemocyte phagocytosis. After rSpCrus8 and rTrx-SpCrus8 treatment, a large number of fluorescent microspheres were observed to aggregate into clusters and be phagocytosed by multiple hemocytes, while hemocytes in the control group phagocytosed only individual microspheres, indicating that SpCrus8 played an important role in opsonization. When the SpCrus8 gene was knocked down, the expression levels of the key phagocytosis-related genes SpRab5 and SpRab7 were significantly downregulated, as well as the IMD signaling pathway genes SpIKKβ and SpRelish, and another crustin gene SpCrus5. Correspondingly, all the SpIKKβ, SpRelish and SpCrus5 genes were significantly upregulated after rSpCrus8 treatment, suggesting that SpCrus8 might be involved in the immunomodulation of *S. paramamosain*. Taken together, this study revealed the immune-related functions of the SpCrus8 gene in opsonization and regulation, which will help us further understand the role of the crustin gene family in the immune system of mud crabs and provide new insights into the function of type II crutins.

## 1 Introduction

Crustin, a typical antimicrobial peptides (AMPs), was first discovered in the hemolymph of shore crabs *Carcinus maenas* since 1999 (1), and it has now become the most abundant class of AMPs in crustaceans, such as the Japanese spiny lobster *Panulirus japonicus* (2), the freshwater prawn *Macrobrachium rosenbergii* (3), the black tiger shrimp *Penaeus monodon* (4), the Pacific shrimp *Litopenaeus vannamei* (5), the hydrothermal vent shrimp *Alvinocaris longirostris* (6), the portunid crab *Portunus pelagicus* (7), the mud crab *Scylla tranquebarica* and *Scylla paramamosain* (8), the Chinese mitten crab *Eriocheir sinensis* (9), the spider crab *Hyas araneus* (10), and several terrestrial isopods (11) etc. In addition to crustaceans, crustin-like sequences also exist in ant genomes (12). Crustins are cationic AMPs, with a molecular weight (MW) of 7-14 kDa and isoelectric points (pI) between 7.0-8.7 (13). They usually have a signal peptide with a length of 16-24 amino acids (aa) at the N-terminus, and a hydrophobic core consisting of 8 cysteine residues forming 4 disulfide bonds at the C-terminus, also known as 4 disulfide cores (DSC), which is structurally similar to the mammalian whey acidic protein domain (WAPD), so crustins are also defined as WAPD-containing proteins. For example, type I, II, and III crustins contain single WAPD (also named single WAPD-containing protein, abbreviated as SWD), and type IV crustins have two WAPDs (named double WAPD-containing protein, abbreviated as DWD). WAPD is considered a critical domain for biological functions, such as exerting antibacterial or protease inhibitory activities (3). In addition to WAPDs, crustins include other regions, such as the cysteine-rich region (CRR) in type I crustins, the glycine-rich region (GRR) and CRR in type II crustins, and the proline and arginine-rich region (PARR) in type III crustins (14).

Crustins are commonly reported to have antimicrobial activity against gram-positive bacteria, gram-negative bacteria, fungi and even viruses *in vitro*, such as Lv-SWD4 (15), SpCrus4 (16), SpCrus5 (17), SpCrus6 (8), crusFpau (18), EsDWD (19), PtCrustin2 (20), and CrustinPm7 (21). They also have good resistance to important clinical pathogens such as *Staphylococcus aureus*, *Escherichia coli*, *Pseudomonas aeruginosa*, and *Candida albicans*, and several common aquatic pathogens including *Aeromonas hydrophila*, *Vibrio parahaemolyticus* and *Vibrio anguillarum*. Some crustin variants, such as CrustinPm1 (21), Fi-Crustin2 (22) are only effective against gram-negative bacteria such as *E. coli*, *Edwardsiella tarda* and *A. hydrophila*, while CrusSp (23), CruFc (24), CrusEs2 (25), CrustinPm1 and CrustinPm4 (4) had only antibacterial activities against gram-positive bacteria, such as *S. aureus*, *Micrococcus luteus* and *Bacillus subtilis*. Exceptionally, several crustins have no obvious antibacterial activity, such as Fc-DWD (26), PcDWD (26), Pc-SWD (27), PmDWD (28), and LvCrustin I-1 (29). However, they have also been confirmed to play an important role in host immunity (e.g., recombinant LvCrustin I-1 could regulate intestinal microbiota homeostasis in *L. vannamei* (29)) and may act as host defense peptides. The *in vivo* immune functions of crustins and the underlying mechanism deserve more attention. In addition to antimicrobial effects, crustins also have the properties of binding, agglutinating, opsonizing and inhibiting protease activity. They can bind to pattern associated membrane pattern molecules (PAMPs), as well as directly bind and agglutinate microorganisms, such as LvCrustinB, and SpCrus6 showed strong binding activity to PAMPs and bacteria. The PcCru recombinant protein of *P. clarkia* and SpCrus2 recombinant protein could agglutinate certain gram-positive and gram-negative bacteria. Recently, a newly reported type VII crustin LvCrustinVII from *L. vannamei* was identified, and its recombinant protein has been proved to agglutinate *V. parahaemolyticus* and enhance hemocyte phagocytosis in the presence of Ca^2+^, indicating that it might be an opsonin (30). Similar aggulutination and opsonization were also found in type I crustin MjCruI-1 from *Marsupenaeus japonicus* (31). Most crustins with protease inhibitory activity are mainly type III and IV crustins, such as LvSWD (32), MjSWD (33), Mj-DWD (34), Fc-DWD (26), and Pc-DWD (35). Taken together, although different custins perform various activities, their roles in host immunity are essential. In addition to the confirmed functions mentioned above, whether they have other roles in assisting the host to eliminate pathogens is worth exploring.

At present, a total of seven crustin genes have been identified in the mud crab *S. paramamosain*, referred to as SpCrustins. Most of them belong to type I crustin, such as CrusSp (23), SpCrus3 (16), SpCrus4 (16), SpCrus6 (8) and SpCrus7 (36). The type II crustins include only SpCrus2 (37) and SpCrus5 (17). The functional study of SpCrustins mainly focused on direct antibacterial activity. Most reported SpCrustins have good antibacterial activity against gram-negative and gram-positive bacteria, and CrusSp and SpCrus7 only showed antibacterial activity against certain gram-positive bacteria. They can also bind to PAMPs and agglutinate microorganisms (except CrusSp and SpCrus7), which may contribute to their antibacterial function. In addition, SpCrus6 could bind to the envelope protein VP26 of WSSV, inhibit the replication of virus and significantly reduce the viral load, proving the antiviral function of SpCrus6 *in vivo* (8). They have all been shown to be involved in the immune response of mud crabs and play an important role in protecting the crabs from infection. Quite a few crustin genes are expected to be discovered in mud crabs, especially type II crustins. Further elucidation of their functions will help us to understand the role of this gene family in the immune system of mud crabs, and provide basic information for disease prevention and control in crab farms.

In this study, based on the transcriptome database of *S. paramamosain* established previously by our research group, a new crustin family member (named SpCrus8) was identified, which was subsequently considered to be type II crustin. After obtaining the full-length cDNA of the SpCrus8 gene by RACE-PCR, the tissue distribution and expression patterns in the context of bacterial infection were comparatively studied by qPCR, and recombinant SpCrus8 proteins (rSpCrus8 and rTrx-SpCrus8) were expressed by a prokaryotic expression system and purified by affinity chromatography. Immune-related functions (such as binding activity, opsonization, immunoprotection, regulation, etc.) of the recombinant proteins were systematically analyzed, and further verified by RNAi technology. This study aimed to understand the role of the SpCrus8 gene in the immune defense of *S. paramamosain*.

## 2 Materials and Methods

### 2.1 Animals, Strains, Sample Collection and Immune Challenge

Adult mud crab *S. paramamosain* were purchased from a farm in Zhangpu County, Fujian Province, China. Bacteria used in the study were purchased from China General Microbiological Culture Collection Center (CGMCC) (including *V. alginolyticus* CGMCC NO. 1.1833, *S. aureus* CGMCC NO. 1.2465 and *P. aeruginosa* CGMCC NO. 1.2421).

Tissues were separately collected from either normal adult male or female mud crabs (300 ± 30 g, n=5), including hemocytes (Hc), subcuticular epidermis (SE), thoracic ganglion (TG), heart (Ht), midgut (Mg), hepatopancreas (Hp), stomach (St), gills (Gi), eyestalk (Es), female crab gonads: ovaries (Ov), spermathecae (Sp), reproductive duct (RD), male crab gonads: testis (T), anterior vas deferens (AVD), seminal vesicle (SV), ejaculatory duct (ED), posterior ejaculatory duct (PED), posterior vas deferens (PVD). All samples were stored at -80°C for later use.

After 5 days of temporary culture in laboratory, 90 male crabs (300 ± 30 g) were randomly selected and divided into 3 groups (30 crabs in each group), including the control group (injection of crabs with saline solution, 100 μL for each crab), *V. alginolyticus* infection group (3×10^6 CFU/mL, 100 μL for each crab) and *S. aureus* infection group (3×10^6 CFU/mL, 100 μL for each crab). The injection was carried out between the third and fourth appendages of the crabs, and hepactopancreas of five individuals were sampled at each time point (3 h, 6 h, 12 h, 24 h, 48 h, and 72 h post infection) for each treatment, and they were set as five biological replicates. The samples were quickly put in liquid nitrogen and then transferred to -80°C for future use. All animal procedures were carried out in strict accordance with the National Institute of Health Guidelines for the Care and Use of Laboratory Animals and were approved by the Animal Welfare and Ethics Committee of Xiamen University.

Total RNA of all collected samples was extracted using Trizol reagent (Thermo Fisher Scientific, USA) according to the manufacturer′s instructions, and then cDNA was synthesized using PrimeScript™ RT reagent kit with gDNA eraser (TaKaRa, Japan).

### 2.2 Cloning of the Full-Length cDNA of SpCrus8 Gene

SpCrus8 gene-specific primers were designed and synthesized by Xiamen Primus Biotechnology Co., Ltd. (Xiamen, China) according to the partial sequence from the transcriptome database established by our research group (listed in **Table S1**). The cDNA of male crab testis was used as the template, and the primers SpCrus8-CDS-F and SpCrus8-CDS-R were used to amplify the coding sequence (CDS) of the SpCrus8 gene. The 5′- and 3′- RACE cDNA template of testis were prepared as described previously (38). Nested PCR was employed to amplify the 5’- and 3’- cDNA end of the SpCrus8 gene. The primers SpCrus8-5’-R1 and SpCrus8-3´-F1 were used in the first round PCR amplification, and SpCrus8-5’-R2 and SpCrus8-3´-F2 were used in the second round PCR amplification, paired with long primer and short primer provided by SMARTer^®^ RACE 5′/3′ Kit (Clontech, USA), respectively (**Table S1**). The touch down PCR procedure was performed as followed: 95 °C, 5 min; 30 cycles of 95 °C, 30 s, 68 °C -0.5/cycle, 30 s, 72°C, 2 min; 72 °C, 10 min; 16 °C 5 min. The PCR products were then purified and sequenced.

### 2.3 Bioinformatics and Phylogenetic Analysis of SpCrus8

Bioinformatics analysis of SpCrus8 gene was carried out using several online websites and some software. For example, SignaIP-5.0 Server (https://services.healthtech.dtu.dk/service.php/SignalP-5.0) was used for signal peptide prediction; protein domain was predicted by SMART database (http://smart.embl-heidelberg.de/smart/list_genomes.pl); Expasy ProtParam tool (https://web.expasy.org/protparam/) was used for protein physicochemical properties analysis. The phylogenetic tree was constructed by MEGA software version 6.0 (using neighbor-joining method, 3000 bootstrap replications) and multiple sequence alignment was conducted by DNAMAN software version 8.0.

### 2.4 Quantitative Real Time PCR (qPCR) Analysis

Absolute qPCR was used to analyze the tissue distribution of the SpCrus8 gene in *S. paramamosain*. And relative qPCR was employed to determine the expression pattern of the SpCrus8 gene in the hepatopancreas of crabs under bacterial challenge (*V. alginolyticus* and *S. aureus*), as well as the expression of several immune-related genes (including SpRab5 (GenBank accession NO. FJ774735), SpRab7, SpIMD (GenBank accession NO. MH047673), SpTAK1 (GenBank accession NO. MK319934), SpIKKβ (GenBank accession NO. MF374338), SpIKKϵ (GenBank accession NO. MF374339), SpRelish (GenBank accession NO. MH047674), SpCrus3 (GenBank accession NO. MF431587), SpCrus4 (GenBank accession NO. MF431588) and SpCrus5 (GenBank accession NO. MF431586)) in primary cultured hemocytes treated with recombinant SpCrus8 or dsRNA for SpCrus8. SpGAPDH gene (GenBank accession NO. KJ133040) was taken as the internal reference gene. The specific primers for genes were listed in **Table S1**. qPCR was carried out on Applied Biosystems 7500 Real-Time PCR system (Applied Biosystems, USA) using FastStart DNA Master SYBR Green I (Roche Diagnostics, Germany), and the PCR procedure was the same as previously reported (38). The absolute copy number of SpCrus8 gene in tissues was obtained according to the standard curve made by serial dilution of SpCrus8 CDS plasmid. The data for relative qPCR was analyzed using the algorithm of the 2^-△△Ct^ method (39).

### 2.5 Expression and Purification of Recombinant SpCrus8

According to the verified SpCrus8 CDS sequence, specific primers including restriction enzyme sites were designed for the construction of prokaryotic recombinant expression vector (listed in **Table S1**). *Nde* I and *Xho* I were selected as restriction enzymes for recombinant protein (rSpCrus8) construction in pET-30a vector (including 6 × His tag), and *EcoR* I and *Xho* I for recombinant protein (rTrx-SpCrus8) construction in pET-32a vetor (including 6 × His tag and thioredoxin (Trx) tag). They were expressed in *E. coli* BL21 (DE3) plus. The optimal conditions for recombinant protein expression were as follows: for rSpCrus8, when the bacterial OD_600_ value reached 0.3-0.4, induced with 0.5 mM IPTG for 9 h at 37°C, or for rTrx-SpCrus8, induced with 0.5 mM IPTG for 4 h at 37°C when the bacterial OD_600_ value reached 0.6. The recombinant proteins (including rSpCrus8, recombinant Trx (rTrx), rTrx-SpCrus8) were then purified by affinity chromatograph using ÄKTA Pure system (GE Healthcare Life Sciences, USA) using a HisTrap™ FF Crude column (GE Healthcare Life Sciences, USA). The purified proteins were analyzed by SDS-PAGE and the target bands were confirmed by the Mass Spectrometry Center of the School of Life Sciences, Xiamen University. The Pierce BCA Protein Assay Kit (Thermo Fisher Scientific, USA) was used to detect the protein concentration.

### 2.6 Binding Assay

The binding ability of SpCrus8 recombinant proteins to microbial surface components, four typical PAMPs including lipopolysaccharide (LPS B5, Sigma, USA), lipoteichoic acid (LTA, L2515, Sigma, USA), peptidoglycan (PGN from *B. subtilis*, Sigma, USA), glucan (GLU, baker’s yeast, Merck, Germany) and several bacteria (including *S. aureus*, *P. aeruginosa* and *V. alginolyticus*) was performed by a modified ELISA assay as previously described (40). Briefly, LPS, LTA, PGN and GLU were diluted to a working concentration of 20 μg/mL with ELISA coating solution (Solarbio, Binjing), and bacteria were cultured to an OD_600_ of 0.6 and suspended with ELISA coating solution to OD_600_ of 1. They were added to a 96-well plate and incubated overnight at 4°C. Subsequently, they were blocked with 5% nonfat milk for 2 h at 37°C. Different concentrations of rSpCrus8 (0-2 μM) were added (for bacterial binding assay, 1 μM rTrx, rTrx-SpCrus8, rSpCrus8 were added) and incubated at 37°C for 2 h. The primary antibody (mouse anti-His antibody, 1:3000, Proteintech, USA) was added followed by incubation with goat anti-mouse HRP antibody (1:5000, Invitrogen, USA). The absorbance at 450 nm was measured using a microplate reader (TECAN GENios, GMI, USA). Data were recorded and analyzed for binding efficiencies [represented by the apparent dissociation constant (Kd)]. Each group had three biological parallels. The independent assay was repeated at least three times.

### 2.7 *In Vivo* Bacterial Clearance Assay and Immunoprotection Assay

Mud crabs *S. paramamosain* (120 g ± 5 g) were temporarily cultured for three days before the experiment, and 100 μL of 5.4×10^7 CFU/mL *V. alginolyticus* was then injected into crabs. The recombinant protein rTrx and rTrx-SpCrus8 was diluted with PBS to 500 μg/mL, and 30 min after bacterial infection, the experimental group was injected with 100 μL of proteins. The control group was injected with an equal volume of PBS. There were four parallels in each group. After additional 30 minutes, 80 μL of hemolymph was taken and plated on 2216 plates overnight, and the bacteria was counted to analyze the bacterial clearance efficiency. The independent assay was repeated at least three times.

In the immunoprotection assay, mud crabs *S. paramamosain* (60 g ± 4 g) were temporarily cultured for three days before the experiment, and injected with 40 μL of 6×10^8 CFU/mL *V. alginolyticus*. One hour after bacterial infection, each crab was injected with a total of 10 μg of protein (rTrx or rTrx-SpCrus8), and the control group was injected with an equal volume of PBS (n=22). The number of dead crabs at different time point in each group was recorded, and the survival curve was drawn with GraphPad Prism 8.3.0 version. The independent assay was repeated at least twice.

### 2.8 Phagocytosis Assay

FluoSpheres^®^ fluorescent microspheres (Molecular Probes^™^, 1.0 μm, Thermo Fisher Scientific, USA) were prepared according to the manufacturer’s instruction. Briefly, hemocytes from *S. paramamosain* were extracted and seeded at 3×10^6 per well on a 96-well cell culture plate in L-15 medium, as previously described (41, 42). Different concentrations of rSpCrus8 (50 μg/mL, 100 μg/mL), rTrx (100 μg/mL) or rTrx-SpCrus8 (100 μg/mL) were added and incubated for 30 min at room temperature, and equal volume of L-15 medium was used as control. Fluorescent microspheres were then added to a final concentration of 1.5×10^7 (the ratio to hemocytes, 1:20). After 45 minutes of incubation, hemocytes were collected and fixed with 2% glutaraldehyde solution for 15 min. DAPI was used for nuclear staining. The samples were observed by a confocal laser scanning microscope (CLSM, Zeiss Lsm 780 NLO, Germany). And the phagocytosis efficiency was determined by flow cytometry (CytoFLEX, Beckman Coulter, USA). The independent assay was repeated three times.

### 2.9 RNAi Assay

The primers (dsSpCrus8-F and dsSpCrus8-R) for SpCrus8 gene dsRNA synthesis were designed using the online tool (https://www.flyrnai.org/cgi-bin/RNAi_find_primers.pl) (listed in **Table S1**). The primers for GFP dsRNA synthesis (dsGFP-F and dsGFP-R) and RNAi efficiency detection (dtSpCrus8-F and dtSpCrus8-R) were also shown in **Table S1**. The dsRNAs of SpCrus8 gene and GFP gene were synthesized using the T7 RiboMAX™ Express RNAi System kit (Promega, USA) according to the manufacturer’s instruction.

Hemocytes were primarily cultured as mentioned above. dsRNA was transfected (final concentration of 3 μg/mL) using Lipofectamine 3000 (Thermo Fisher Scientific, USA) according to the manufacturer’s instruction, and 24 hours later, dsRNA was transfected again following the same procedure. After another 24 hours, samples were collected and the total RNA was extracted for subsequent analysis. RNAi efficiency was detected by semi-quantitative PCR and the optical density of target bands was analyzed on the ImageJ website (https://ij.imjoy.io/). The independent assay was repeated at least three times. After dsRNA treatment, phagocytosis assay was performed and the expression levels of several immune-related genes (including SpRab5, SpRab7, SpIMD, SpTAK1, SpIKKβ, SpIKKϵ, SpRelish, SpCrus3, SpCrus4 and SpCrus5) were analyzed using relative qPCR as mentioned above.

### 2.10 Statistical Analysis

All data were presented as mean ± standard error of the mean (SEM). All the statistical analyses were performed using GraphPad Prism 8.3.0 version. For absolute qPCR, one-way ANOVA was employed to compare differences of SpCrus8 gene expression in different tissues. For relative qPCR and binding assay, a method of multiple t test (one per row) was used. In phagocytosis assay and bacterial clearance assay, control and experimental groups were compared using unpaired t-test with Welch’s correction. For immunoprotection assay, survival comparisons were analyzed using the Kaplan-Meier Log rank test. Difference was considered as significant at p value< 0.05.

## 3 Results

### 3.1 Sequence and Structure Analysis of SpCrus8

**Figure 1:**
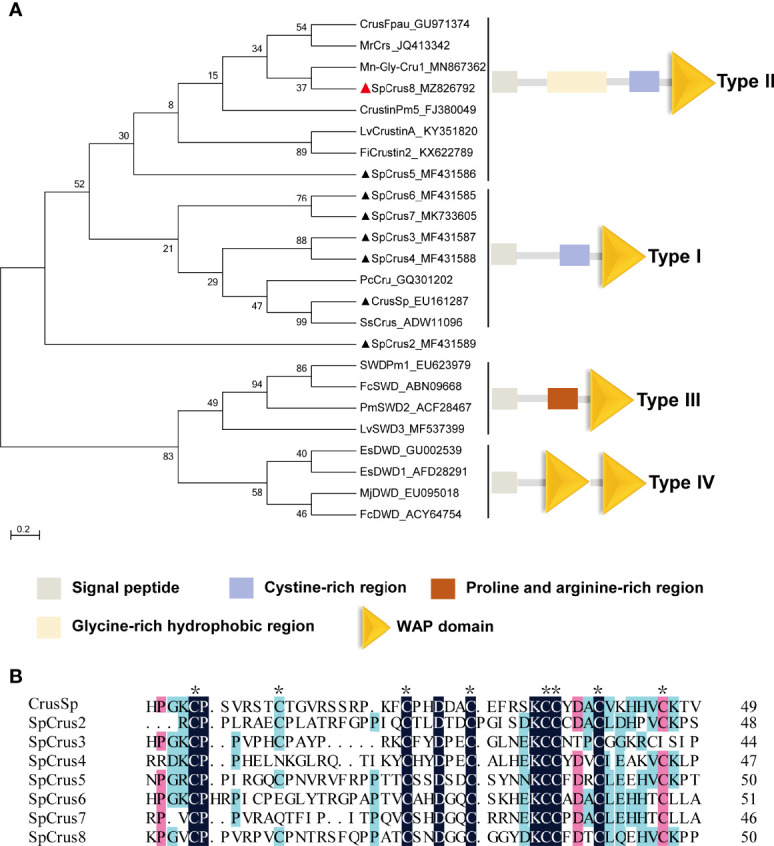
Phylogenetic analysis of SpCrus8 and multiple sequence alignment of SpCrustins. **(A)** Phylogenetic tree analysis of SpCrus8. A neighbor-joining phylogenetic tree of crustins was constructed using MEGA ver.6.0 software with 3000 bootstrap replications. SpCrus8 is marked with a red triangle and other crustins of *S. paramamosain* are marked with black triangles. **(B)** Multiple sequence alignment of WAPD of crustins from *S. paramamosain*. Eight conserved cysteines involved in the formation of the disulfide bridges in WAPD is indicated by “*”. Similar amino acids are shaded in red, light blue or dark blue. The information of the sequences is listed as follows: Mn: *Macrobrachium nipponense*; Sp: *Scylla paramamosain*; Lv: *Litopenaeus vannamei*; Pm: *Penaeus monodon*; Pj: *Panulirus japonicus*; Fi: *Fenneropenaeus indicus*; Mj: *Marsupenaeus japonicus*; Fc: *Fenneropenaeus chinensis*; Pc: *Procambarus clarkii*; Ss: *Scylla serrata*. CrusSp (GenBank: EU161287), SpCrus2 (GenBank: MF431589), SpCrus3 (GenBank: MF431587), SpCrus4 (GenBank: MF431588), SpCrus5 (GenBank: MF431586), SpCrus6 (GenBank: MF431585), SpCrus7 (GenBank: MK7336055).

The full-length cDNA sequence of the new crustin homolog SpCrus8 gene (GenBank accession NO. MZ826792) was obtained, and was 992 bp in length, including a 159 bp 5’ untranslated region (UTR), a 155 bp 3’ UTR, and a 678 bp open reading frame (**Figure S1A**). The gene is expected to encode a total of 225 amino acids (aa) with a MW of 20.6 kDa, including a 27 aa signal peptide, and the pI of the mature peptide is 7.77. In addition, it contains a GRR, a CRR and a WAPD (**Figures S1A, B**), which are structurally similar to those of type II crustins. Phylogenetic tree analysis further confirmed that the SpCrus8 gene shares the same branch as type II crustins (**Figure 1A**).

The CDS of the SpCrus8 gene had 38.86%-55.56% amino acid identity with other crustins identified from *S. paramamosain*, with the highest similarity (55.56%) with SpCrus5 (**Table S2**). As shown in **Table S2** and **Figure 1B**, WAPD is evolutionarily highly conserved, with a similarity of 46.81%-63.27% among SpCrustins, and it contains 8 cysteines that might form four disulfide bonds.

### 3.2 Expression Profiles of the SpCrus8 Gene

**Figure 2:**
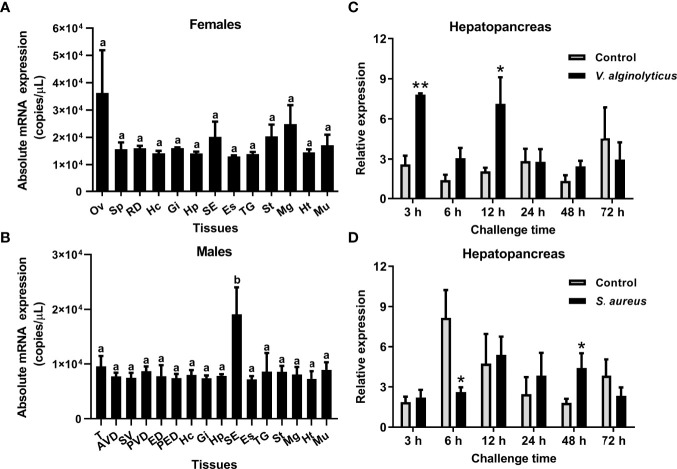
Gene expression profiles of SpCrus8 in *S. paramamosain*. Tissue distribution of SpCrus8 in females **(A)** and males **(B)** crabs (n=5). The expression pattern of SpCrus8 in male hepatopancreas after *V. alginolyticus* **(C)** and *S. aureus* **(D)** challenge (n=5). Differences in different tissues were indicated with the letter “a” or “b”. Significant difference between the control and bacterial challenge groups was indicated with asterisks, *p < 0.05, **p < 0.01. Abbreviations: Ov, ovaries; Sp: spermathecae; RD, reproductive duct; Hc, hemocytes; Gi, gills; Hp, hepatopancreas; SE, subcuticular epidermis; Es, eyestalk; TG, thoracic ganglion; St, stomach; Mg, midgut; Ht, heart; Mu, muscle; T, testis; AVD, anterior vas deferens; SV, seminal vesicle; PVD, posterior vas deferens; ED, ejaculatory duct; PED, posterior ejaculatory duct.

The tissue distribution of the SpCrus8 gene, as well as the gene expression pattern in the hepatopancreas of *S. paramamosain* under bacterial challenge were investigated. The results showed that the SpCrus8 gene was widely distributed in the tissues of female and male crabs, with the highest expression level in the ovaries of females (average of 3.62×10^4 copies/μL), and the subcuticular epidermis of males (average of 1.91×10^4 copies/μL, significantly different from other tissues) (**Figures 2A, B**).

In the hepatopancreas of crabs infected with *V. alginolyticus*, transcripts of the SpCrus8 gene were significantly increased at 3 h and 12 h in the early stage of infection, and then returned to a level similar to that of the control group (**Figure 2C**). When the crabs were challenged with *S. aureus*, SpCrus8 gene expression was significantly downregulated at 6 h and upregulated at 48 h (**Figure 2D**).

### 3.3 Protein Activity of Recombinant SpCrus8 *In Vitro* and *In Vivo*

**Figure 3:**
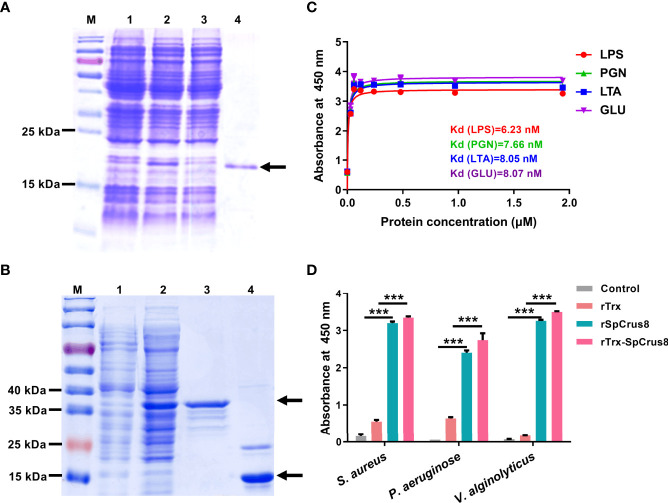
Recombinant SpCrus8 expression and binding activity. Expression and purification of recombinant SpCrus8. **(A)** Lane M: protein molecular standard; lane 1: *E*. *coli* BL21 (DE3) containing recombinant vector (pET-30a-SpCrus8) before induction; lane 2: *E*. *coli* BL21 (DE3) containing recombinant vector (pET-30a-SpCrus8) after induction by 0.5 mM isopropyl-β-D-thiogalactopyranoside (IPTG); lane 3: flow-through during protein purification; lane 4: purified rSpCrus8. **(B)** Lane M: protein molecular standard; lane 1: *E*. *coli* BL21 (DE3) containing recombinant vector (pET-32a-SpCrus8) before induction; lane 2: *E*. *coli* BL21 (DE3) containing recombinant vector (pET-32a-SpCrus8) after induction by 0.5 mM IPTG; lane 3: purified rTrx-SpCrus8. lane 4: purified rTrx. **(C)** Binding activity of rSpCrus8 to PAMPs (LTA for lipoteichoic acid, LPS for lipopolysaccharide, PGN for peptidoglycan, GLU for glucan). **(D)** Binding activity of rSpCrus8, rTrx-SpCrus8 and rTrx to bacteria (*S. aureus*, *P. aeruginosa* and *V. alginolyticus*) and PBS was used as negative control. Significant difference was indicated with asterisks, ***p < 0.001.

Recombinant proteins including rSpCrus8 (**Figure 3A**), rTrx and rTrx-SpCrus8 (**Figure 3B**) were successfully obtained from the prokaryotic expression system and confirmed by mass spectrometry (**Figure S2**). The binding activity and opsonization of the recombinant proteins *in vitro* and the bacterial clearance ability and immunoprotective effect *in vivo* were further analyzed. Since the yields of rSpCrus8 and rTrx-SpCrus8 were different (0.385 mg/L and 3.23 mg/L, respectively), in the *in vivo* assay, rTrx-SpCrus8 was used.

### 3.4 Binding Activity

The binding ability of recombinant SpCrus8 to four PAMPs and bacteria was detected by ELISA. As shown in **Figure 3C**, rSpCrus8 could obviously bind to LTA, LPS, PGN and GLU. The Kd values were 6.23 nM for LPS, 7.66 nM for PGN, 8.05 nM for LTA, and 8.07 nM for GLU. rTrx-SpCrus8 also showed similar binding activity to four PAMPs as rSpCrus8 (**Figure S3**). In addition, rSpCrus8 and rTrx-SpCrus8 exhibited significant binding affinity to the tested bacteria (*S. aureus*, *P. aeruginosa* and *V. alginolyticus*) (**Figure 3D**). The results suggested that the recombinant proteins of SpCrus8 had strong binding activity not only to microbial surface components, but also to bacteria.

### 3.5 Opsonization

**Figure 4:**
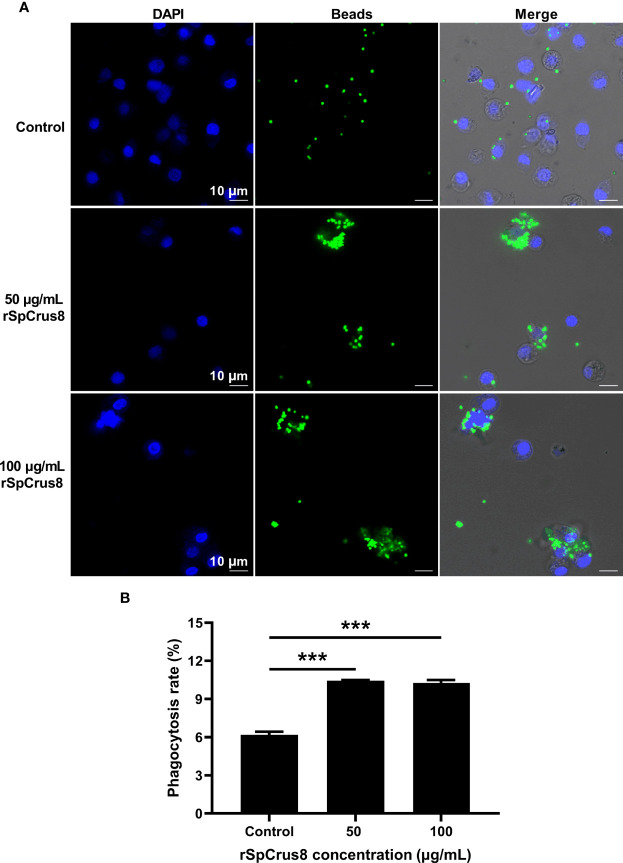
Phagocytosis of fluorescent microspheres by hemocytes after rSpCrus8 treatment. **(A)** Phagocytosis of fluorescent microspheres by hemocytes after rSpCrus8 treatment (50 µg/mL or 100 µg/mL) was observed under CLSM, and equal volumn of L-15 medium was used as control. The nuclei of hemocytes were stained with 4,6-diamino-2-phenyl-indole (DAPI) (blue), and green fluorescence represented fluorescent microspheres. **(B)** The phagocytosis rate of fluorescent microspheres by hemocytes was determined by flow cytometry. Significant difference was indicated with asterisks, ***p < 0.001.

The effect of rSpCrus8 and rTrx-SpCrus8 treatment on crab hemocyte phagocytosis was observed by confocal fluorescence microscopy and analyzed by flow cytometry. Compared with the control group, rSpCrus8 significantly promoted the phagocytosis of fluorescent microspheres. The phagocytosis rates of the control group, and the 50 μg/mL and 100 μg/mL rSpCrus8 treatment groups were 6.18%, 10.44%, and 10.27%, respectively (**Figure 4B**). Likewise, the phagocytosis rate of the rTrx-SpCrus8 treatment group was significantly increased by 15.7% and 23.6% compared with the control groups (PBS and rTrx group) (**Figure S4B**). After rSpCrus8 and rTrx-SpCrus8 treatment, a large number of fluorescent microspheres were observed to aggregate into clusters and be phagocytosed by multiple hemocytes (formed aggregates of hemocytes and microspheres, similar to hemocyte nodulation), while in the control group, most hemocytes only phagocytosed individual microspheres (**Figure 4A** and **Figure S4A**).

### 3.6 Bacterial Clearance Ability and Immunoprotective Effect *In Vivo*

**Figure 5:**
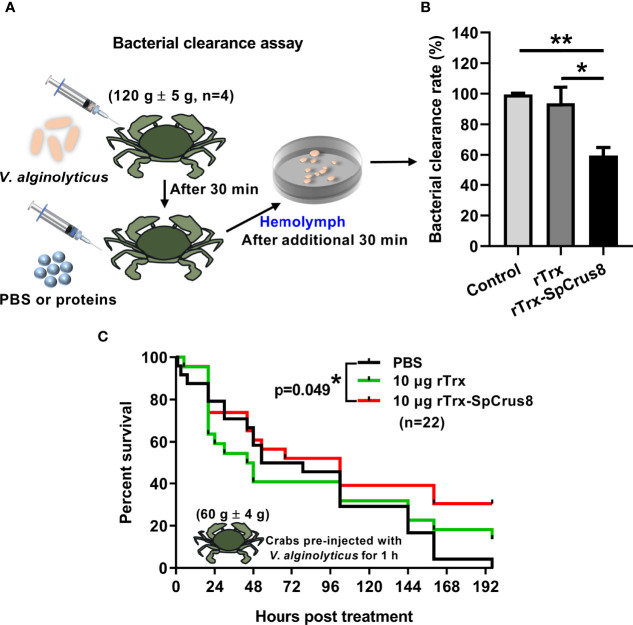
Effects of rTrx-SpCrus8 treatment on hemolymph bacterial clearance and immune protection in crabs infected with *V. alginolyticus*. **(A)** Schematic diagram of the bacterial clearance experiment in *S. paramamosain*. **(B)** The bacterial clearance rate of *V. alginolyticus* in hemolymph after rTrx-SpCrus8 or rTrx treatment *in vivo* (n=4). **(C)** The survival curve of mud crabs infected with *V. alginolyticus* after rTrx-SpCrus8 or rTrx treatment. Male crabs were challenged with *V. alginolyticus*, and injected with rTrx-SpCrus8 or rTrx (10 μg crab^-1^) 1 h after bacterial challenge (n=22). Significant difference was indicated with asterisks, *p<0.05 and **p < 0.01.

Since the recombinant proteins of SpCrus8 had a strong binding affinity for PAMPs and bacteria and played an important role in hemocyte opsonization *in vitro*, it was worth investigating the effect of SpCrus8 on the bacterial clearance ability of crab hemolymph and the immunoprotection of crabs infected with *V. alginolyticus in vivo*. The results showed that the bacterial load in the hemolymph of crabs after rTrx-SpCrus8 treatment was significantly reduced by 40% (p=0.004) or 34% (p=0.037) compared to the PBS group or the rTrx group, respectively (**Figures 5A, B**).

In the immunoprotection assay, compared with the PBS group, treatment of 10 μg rTrx-SpCrus8 significantly improved the survival rate of mud crabs infected with *V. alginolyticus* (**Figure 5C**). After 196 h of treatment, the survival rates of the PBS, rTrx and rTrx-SpCrus8 groups were 0%, 13.6%, and 30.4%, respectively.

### 3.7 An RNAi Assay Further Confirmed the Opsonization Role of the SpCrus8 Gene

**Figure 6:**
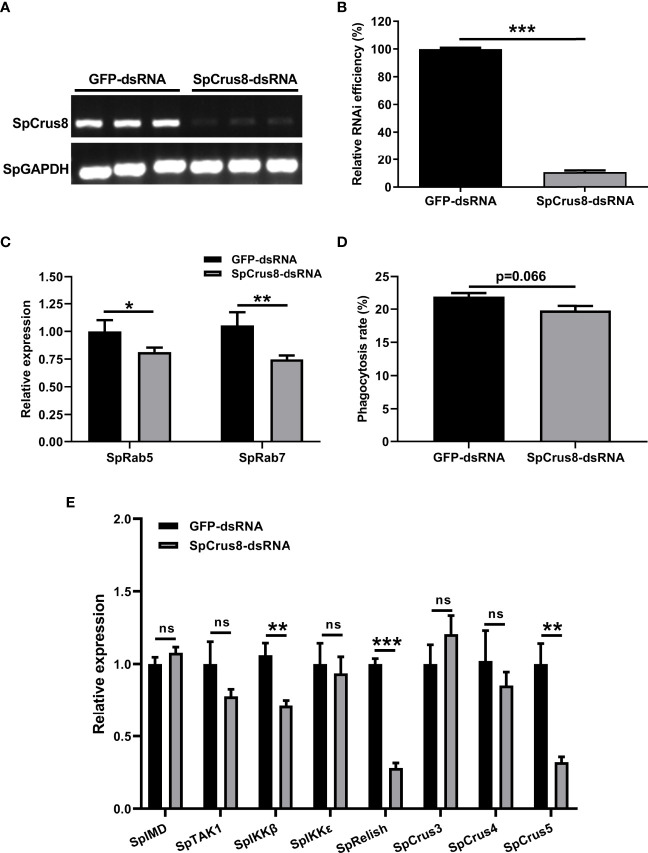
Effect of SpCrus8 gene knockdown on phagocytosis rate and immune-related gene expression in hemocytes. The RNA interference efficiency of SpCrus8 was detected by RT-PCR **(A)** and analyzed by ImageJ **(B)**, and SpGAPDH was used as the internal reference. **(C)** The expression of phagocytosis-related genes (SpRab5 and SpRab7) in hemocytes were examined by qPCR (n=3). **(D)** The phagocytosis rate of fluorescent microspheres by hemocytes after SpCrus8-dsRNA or GFP-dsRNA treatment was determined by flow cytometry. **(E)** The expression of immune-related genes (SpIMD, SpTAK1, SpIKKβ, SpIKKϵ, SpRelish, SpCrus3,SpCrus4 and SpCrus5) in hemocytes were examined (n=3) after being treated with SpCrus8-dsRNA. Significant difference between SpCrus8-dsRNA treatment group and GFP-dsRNA group was indicated with asterisks, *p < 0.05, **p < 0.01, and ***p < 0.001, and ns represented no significant difference.

An RNAi assay was conducted to reveal the immune-related function of SpCrus8 gene. The results showed that the relative interference efficiency was approximately 89% (**Figures 6A, B**), indicating that the SpCrus8 gene was significantly inhibited in crab hemocytes. Compared with the GFP-dsRNA group, the phagocytosis rate of the hemocytes in the SpCrus8-dsRNA group was reduced (**Figure 6D**), but there was no significant difference (p=0.066). It was interesting to note that the expression levels of the key phagocytosis-related genes SpRab5 and SpRab7 were significantly decreased in the SpCrus8-dsRNA group (**Figure 6C**).

### 3.8 The SpCrus8 Gene Might Play a Role in the Regulation of SpIKKβ, SpRelish and SpCrus5

**Figure 7:**
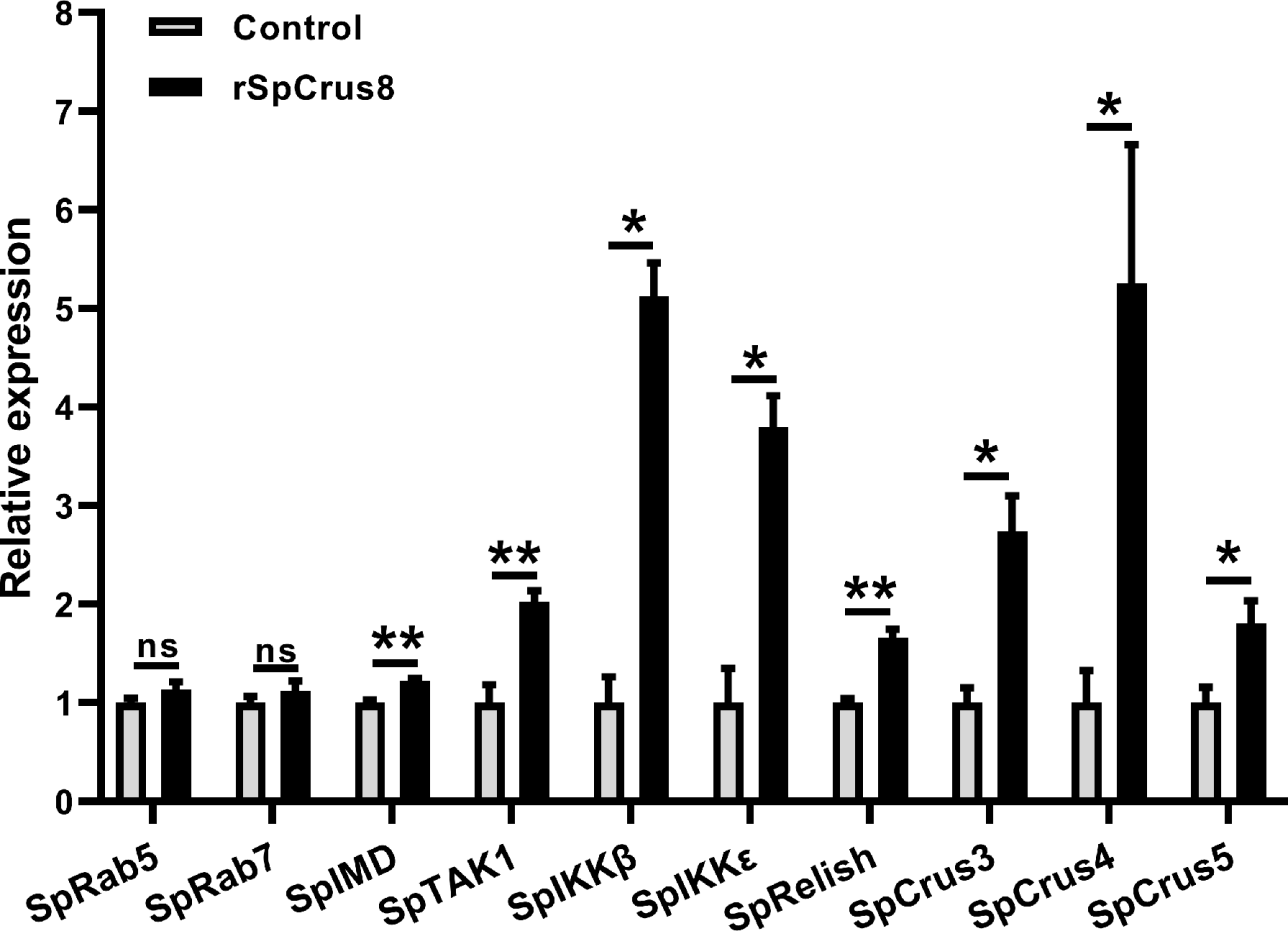
The regulatory role of SpCrus8 in hemocytes of *S. paramamosain*. The expression of immune-related genes (SpRab5, SpRab7, SpIMD, SpTAK1, SpIKKβ, SpIKKϵ, SpRelish, SpCrus3,SpCrus4 and SpCrus5) in hemocytes were examined (n=3) after being treated with rSpCrus8. Significant difference was indicated with asterisks, *p < 0.05 and, **p < 0.01 and ns represented no significant difference.

To further understand the regulatory role of the SpCrus8 gene in *S. paramamosain*, the expression patterns of the IMD signaling pathway genes SpIMD, SpTAK1, SpIKKβ, SpIKKϵ, SpRelish and other crustin genes (SpCrus3, SpCrus4, and SpCrus5) were analyzed after SpCrus8-dsRNA treatment or rSpCrus8 treatment. The expression levels of the SpIKKβ, SpRelish and SpCrus5 genes were significantly downregulated after the SpCrus8 gene was knocked down (**Figure 6E**). Correspondingly, all these three genes were remarkably upregulated after rSpCrus8 treatment (**Figure 7**). In addition, SpIMD, SpTAK1, SpIKKϵ, SpCrus3 and SpCrus4 gene expression were also significantly induced in the rSpCrus8 treatment group (**Figure 7**). These results suggested that the SpCrus8 gene might be involved in the immunomodulation of *S. paramamosain*.

## 4 Discussion

Antimicrobial peptides (AMPs), also known as host defense peptides, in addition to broad-spectrum antibacterial activities, also have other biological functions, including but not limited to antifungal, antiviral, antitumor, antiparasitic activity, modulating host immunity, and promoting wound healing (43–45). Crustins are abundant classical AMPs in crustaceans and play an important role in immune defense, and some of which have direct antibacterial and antiviral activities [such as SpCrus6 (8), crusFpau (18), EsDWD (19), and PtCrustin2 (20)]. However, a considerable number of crustins were found to have weak antibacterial activity *in vitro*, but significant immune protection *in vivo* [such as Pc-SWD (27), and PmDWD (28)]. The specific mechanism is still unclear, and deserves further in-depth study. In this study, we obtained a new member of the crustin gene family, SpCrus8, from *S. paramamosain*. The gene structure of SpCrus8 contains CRR, GRR and WAPD, which is in line with the typical characteristics of type II crustins. SpCrus2 and SpCrus5 in mud crabs also belong to the same type as SpCrus8, and their WAPD amino acid sequences share 52.17% and 63.27% similarity with that of SpCrus8, respectively. It was found that the SpCrus8 recombinant protein had no obvious antibacterial activity, but could significantly promote the hemocyte phagocytosis, enhance the bacterial clearance ability of hemolymph and improve the survival rate of crabs infected by *V. alginolyticus*. This study aimed to initially reveal the underlying molecular mechanism.

First, the *in vivo* expression profiles of the SpCrus8 gene were analyzed. The gene was widely distributed in various tissues of male and female crabs. The highest expression in male crabs was the subcuticular epidermis. The tissues with high expression levels of crustacean crustins are mostly hemocytes, gills, intestines and stomachs. Of the crustins in *S. paramamosain*, except for CruSp, which was most expressed in the hemocytes, SpCrus2-6 were highly expressed in the gills (8, 17, 46). Additionally, it is noted that most crustins in shrimp were identified from hemocytes, such as SWDPm1 (47), Pc-crustin 4 (8), Pc-DWD (35), Fi-crustin (22), and LvSWD3 (48). The expression levels of PtCrustin2 and 3 from *Portunus trituratus* was highest in the eye stalk, followed by the gills (20). Type II crustins, such as LvCrustinB (49), LvCrustinA (50), LvCruU (51), Hoa-crustin (52), and crustinPm5 (53), were mainly distributed in the subcuticular epidermis, which is consistent with the high expression of SpCrus8 in male crabs. In addition, the SpCrus8 gene was expressed at moderate levels in immune-related tissues (such as the hepatopancreas and hemocytes).

In crustaceans, the hepatopancreas and hemocytes are key tissues for the production of immune-related factors (54). In this study, the hepatopancreas was selected to analyze the induced expression pattern of the SpCrus8 gene after bacterial infection. Type II crustins have been confirmed to exhibit potent antibacterial activity *in vitro*. Moreover, it was confirmed that this type of crustin could exert anti-gram-negative bacteria and antiviral functions *in vivo*, such as the MjCRS gene from kuruma shrimp *M. japonicus* (55). In this study, it was found that in the hepatopancreas of crabs infected with *S. aureus* or *V. alginolyticus*, the expression of the SpCrus8 gene was significantly regulated, indicating that the SpCrus8 gene was involved in the immune response process of *S. paramamosain* to bacteria. Interestingly, the SpCrus8 gene responded differently to *S. aureus* and *V. alginolyticus*. The expression pattern of the SpCrus8 gene was similar to that of the SWD gene from *L. vannamei*, and they were both significantly upregulated at the early stages of 3 h and 6 h after *V. alginolyticus* infection (56). However, in response to *S. aureus* infection, SpCrus8 showed a pattern of first downregulation and then upregulation. In *M. japonicus*, the expression levels of MjCrus I-4 and MjCrus I-5 in the gills (57), and SWDPm1 identified from the hemocytes of the black tiger shrimp *P. monodon* also showed similar trends after *S. aureus* infection (47), which might be due to early bacterial infection strategies and late activation of host defenses. The above results indicated that the antibacterial effects of SpCrus8 were different *in vivo*.

To understand the immune function of the SpCrus8 gene in *S. paramamosain*, recombinant proteins (rSpCrus8 and rTrx-SpCrus8) were obtained by prokaryotic expression system. Neither protein was found to have significant antibacterial activity (**Table S3**). We speculated that SpCrus8 might play a role in the immune response process through other indirect bactericidal functions. Therefore, we first analyzed the binding affinity of rSpCrus8 to the four PAMPs (LPS, LTA, PGN, and GLU). The results showed that all Kd values were less than 10 nM, and the interaction strength was considered strong when Kd < 100 nM (58), indicating that rSpCrus8 has a high affinity for them. Meanwhile, rTrx-SpCrus8 also showed considerable binding activity to PAMPs, and both proteins could bind to several bacteria (*S. aureus*, *P. aeruginosa* and *V. alginolyticus*). In *S. paramamosain*, SpCrus6 also has strong binding activity to LPS, PGN, GLU and LTA and bacteria *B. subtilis*, *V. harveyi*, *V. parahaemolyticus* and *V. alginolyticus* (8). SpCrus2 has high binding affinity for LTA, LPS, GLU and bacteria (such as *S. aureus*, *B. subtilis, V. harveyi*, *V. parahaemolyticus* and *V. alginolyticus*), but weak binding activity to PGN derived from *S. aureus* (37). LvCrustinB from *L. vannamei* can bind to PGN, LTA and LPS (49); MjSWD from *M*. *japonicus* has strong binding activity to PGN, but weaker binding activity to LPS and LTA (33). Different crustins have different binding activities to microbial cell surface components, which may be due to the different physicochemical properties of custins. Whether the strong binding of rSpCrus8 to PAMPs is related to the activation of downstream signaling pathways and is involved in immunomodulation of *S. paramamosain* deserves further exploration.

In crustaceans, phagocytosis plays an important role in cellular immunity, which is mainly mediated by hemocytes. Crustins have also been reported to be involved in the regulation of hemocyte phagocytosis, such as type VII crustin LvCrustinVII from *Litopenaeus vannamei* (30) and type I crustin MjCruI-1 from *Marsupenaeus japonicus* (31). Both could agglutinate bacteria and promote hemocyte phagocytosis, and for LvCrustinVII, its activity became more pronounced in the presence of Ca^2+^. MjCruI-1 had no antibacterial activity against *V. anguillarum* and *S. aureus in vitro*, but when MjCruI-1 was coincubated with FITC-labeled *V. anguillarum* and *S. aureus* for a period of time, the mixture was injected into shrimp. The results showed that compared with the control group, the phagocytosis rate of the MjCru I-1 group was significantly increased, indicating that the potential immune function of MjCru I-1 was related to the phagocytosis of hemocytes (31). In this study, to explore whether SpCrus8 gene was also involved in the phagocytosis of *S. paramamosain* hemocytes, fluorescent microspheres were used which is an ideal material for analyzing phagocytosis (59). The results showed that 50 μg/mL rSpCrus8 could effectively promote the phagocytosis of fluorescent microspheres by hemocytes. In the SpCrus8-dsRNA treatment group (SpCrus8 gene expression in hemocytes was effectively knocked down), the transcription levels of phagocytosis-related genes SpRab5 and SpRab7 (Rab5 is distributed in early phagosomes and rapidly recruits phagosomes, followed by Rab7 on mature phagosome membranes (60)) were significantly reduced, and the phagocytosis rate of hemocytes had a downward trend compared with the GFP-dsRNA group, but there was no significant difference. The suppressed expression of the SpCrus8 gene might affect the expression of other immune factors. To maintain homeostasis, hemocytes might adopt a compensatory phagocytic mechanism when SpCrus8-mediated phagocytosis is inhibited. The above results preliminarily revealed that the SpCrus8 gene might be involved in the process of phagocytosis of pathogens by hemocytes. Since it can efficiently bind to microbial cell surface components and bacteria, it might act as an opsonin to recognize foreign pathogens and promote phagocytosis. The specific molecular mechanism needs further investigation.

As mentioned earlier, hemocytes play roles in phagocytosis, nodule formation and encapsulation in cellular immunity, secrete immune effectors, such as AMPs in humoral immunity, and participate in the melanization of the proPO system to effectively eliminate foreign microorganisms. This study demonstrated *in vitro* that SpCrus8 recombinant proteins could promote the phagocytosis of fluorescent microspheres by hemocytes, and showed similar nodule formation. To explore the immune-related function of SpCrus8 recombinant protein in mud crabs, we preinjected *V. alginolyticus* into the crabs for 30 minutes and then injected them with rTrx-SpCrus8. After an additional 30 minutes, the bacterial load in hemocytes in the rTrx-SpCrus8 treatment group was significantly lower than those in the PBS and rTrx groups by 40% and 34%, indicating that rTrx-SpCrus8 could enhance the clearance ability of the mud crab hemolymph against *V. alginolyticus*. The immunoprotection assay further demonstrated that after 196 h, when all the crabs in the PBS group had died, 30.4% of the crabs in the rTrx-SpCrus8 treatment group were still alive, while the survival rate in the rTrx group was only 13.6%, indicating that rTrx-SpCrus8 had a protective effect on *S. paramamosain* infected with *V. alginolyticus*. The mechanism of this effect might be related to the fact that the SpCrus8 recombinant protein promoted the phagocytosis of *V. alginolyticus* by hemocytes. Whether it could simultaneously regulate the expression of some immune effectors or activate the related signaling pathways remains to be further revealed. In recent years, many reported crustins have also been explored for their relevant immune roles *in vivo*. For example, preinjection of MjCruI-1 recombinant protein into *M. japonicus* for 1 h could significantly improve the survival rate of shrimp infected with *V. alginolyticus* or *S. aureus* (31). The LvCrustinB recombinant protein was fed to *L*. *vannamei* for 1 week, and then the shrimp were infected with *V. parahaemolyticus*. The results showed that LvCrustinB recombinant protein feeding could significantly increase the survival rate of shrimp (49). *V. alginolyticus*, a gram-negative bacteria, can cause vibrosis in different aquatic animals, such as shrimp and crabs, as well as humans, posing a huge threat to public health and causing significant economic losses to the aquaculture industry (61). In this study, rTrx-SpCrus8 promoted the bacterial clearance ability of hemocytes to *V. alginolyticus* and reduced the mortality rate of crabs infected with *V. alginolyticus*, indicating that the SpCrus8 gene might be involved in the immune defense process of *S. paramamosain* and has potential application prospects in crab aquaculture.

To further elucidate the immunomodulatory role of the SpCrus8 gene in the innate immunity of *S. paramamosain*, the expression levels of immune-related genes in hemocytes after SpCrus8-dsRNA or rSpCrus8 treatment were analyzed. Interestingly, the SpIKKβ, SpRelish and SpCrus5 genes were found to be significantly upregulated after rSpCrus8 treatment. Correspondingly, after knocking down the SpCrus8 gene, the expression levels of the three genes were remarkably downregulated, indicating that the SpCrus8 gene might play a role in the immune regulation of *S. paramamosain* by affecting the expression levels of the SpIKKβ, SpRelish and SpCrus5 genes.

The activation of AMPs in innate immunity are usually modulated by signaling pathways (such as the Toll and IMD pathways). In the IMD signaling pathway, IMD, TAK, IKK, Relish, etc. are all key factors, which play a key role in the process of resisting pathogen invasion. It was found that the SpIMD, SpIKKs, and SpRelish genes of *S. paramamosain* were significantly upregulated under *V. alginolyticus* infection (62). When the SpIMD gene was knocked down, the downstream pathway genes SpIKKβ, SpRelish and several AMPs including SpALF1-6 and SpCrustin were significantly downregulated; after the expression of SpIKKβ and SpIKKϵ was inhibited, the expression levels of ALFs and crustin were significantly decreased, indicating that SpIMD and SpIKKs may be involved in the regulation of SpALFs and SpCrustins (62). Additionally, it was found that the expression levels of immune-related genes in the hemocytes such as STAT, CrusSp, C-type lectin, proPO, and TLRs were significantly downregulated after relish gene expression was suppressed, the total amount of hemocytes was reduced, two immune parameters, PO and superoxide dismutase activity, were inhibited, and the mortality rate of mud crabs infected with *V. alginolyticus* and WSSV significantly increased, indicating that relish plays a key role in the immune response to *S. paramamosain* (63). In this study, the expression levels of SpIMD, SpTAK1, SpIKKβ, SpIKKϵ, SpRelish and SpCrus3-5 in the hemocytes were all significantly up-regulated after rSpCrus8 treatment, indicating that rSpCrus8 might participate in the activation of the IMD pathway in *S. paramamosain*.

Both the SpCrus5 and SpCrus8 genes are type II crustins, and SpCrus8 has the highest homology with SpCrus5 (which has 55.56% amino acid sequence identity to the CDS of SpCrus8). SpCrus5 recombinant protein showed potent antibacterial activity against a variety of bacteria, such as *S. aureus* (minimal inhibition concentration (MIC) value <0.4 μM), *V. parahaemolyticus* (MIC value <12.5 μM) and *V. harveyi* (MIC value <25 μM), *C. albicans* (MIC value <1.6 μM), had strong binding activity to bacterial cell surface components LPS, PGN and GLU and bacteria *V. alginolyticus*, *V. parahaemolyticus*, *V. harveyi* and *B. subtilis*, and the ability to agglutinate *S. aureus* (17). In this study, both rSpCrus8 and SpCrus8-dsRNA treatment affected the expression of SpIKKβ, SpRelish and SpCrus5 genes, indicating that SpCrus8 might regulate the expression of SpCrus5 and even other immune-related factors by enhancing the expression of SpIKKβ, SpRelish in *S. paramamosain*. The upregulation of SpCrus5 might jointly play a role in antibacterial or immunomodulatory processes, thereby promoting the removal of invading bacteria in hemocytes and improving the survival rate of *S. paramamosain* after *V. alginolyticus* infection. However, the underlying immunomodulatory mechanism of SpCrus8 remains to be further explored.

## Data Availability Statement

The original contributions presented in the study are included in the article/**Supplementary Material**. Further inquiries can be directed to the corresponding author.

## Author Contributions

MJ: data curation, formal analysis, investigation, and methodology; RC: investigation and methodology; XZ: methodology; FC: conceptualization, funding acquisition, and project administration, supervision, writing - original draft, and writing - review editing; K-JW: funding acquisition, project administration, writing - review and editing. All authors contributed to the article and approved the submitted version.

## Funding

This study was supported by the National Natural Science Foundation of Fujian Province, China (grant #2021J05008); the National Natural Science Foundation of China (grant #U1805233); Marine Biotechnology Economic Integration Service Platform from Fujian Association for Science and Technology; the Xiamen Ocean and Fishery Development Special Fund Project (grant #20CZP011HJ06) from the Xiamen Municipal Bureau of Ocean Development; a grant (grant #3502Z20203012) from the Xiamen Science and Technology Planning Project.

## Conflict of Interest

The authors declare that the research was conducted in the absence of any commercial or financial relationships that could be construed as a potential conflict of interest.

## Publisher’s Note

All claims expressed in this article are solely those of the authors and do not necessarily represent those of their affiliated organizations, or those of the publisher, the editors and the reviewers. Any product that may be evaluated in this article, or claim that may be made by its manufacturer, is not guaranteed or endorsed by the publisher.
