# Supplementary material for: A New Crustin Gene Homolog SpCrus8 Identified in Scylla paramamosain Exerting In Vivo Protection Through Opsonization and Immunomodulation
Source: Front Immunol. 2022 Jul 8;13:946227. doi: 10.3389/fimmu.2022.946227 (PMC9305162; doi:10.3389/fimmu.2022.946227)
Supplement: Supplementary file 1 [file DataSheet_1.docx]

**Supplementary materials**

**For**

**A new crustin gene homolog SpCrus8 identified in *Scylla paramamosain* exerting *in vivo* protection through opsonization and immunomodulation**

Manyu Jiang^1^, Roushi Chen^1^, Fangyi Chen^1,2,3^*, Xuewu Zhu^1^, Ke-Jian Wang^1,2,3^

1 State Key Laboratory of Marine Environmental Science, College of Ocean & Earth Sciences, Xiamen University, Xiamen, Fujian, China

2 State-Province Joint Engineering Laboratory of Marine Bioproducts and Technology, College of Ocean & Earth Sciences, Xiamen University, Xiamen, Fujian, China

3 Fujian Innovation Research Institute for Marine Biological Antimicrobial Peptide Industrial Technology, College of Ocean & Earth Sciences, Xiamen University, Xiamen, Fujian, China

*Corresponding author: Fangyi Chen

College of Ocean & Earth Sciences, Xiamen University, Xiamen, Fujian 361102, PR China.

E-mail: chenfangyi@xmu.edu.cn (F. Chen)

**Antibacterial activity assay**

The strains, including *Staphylococcus aureus* (CGMCC1.2465), *Staphylococcus epidermidis* (CGMCC1.4260), *Listeria onocytogenes* (CGMCC1.10753), *Enterococcus faecalis* (CGMCC1.2135), *Corynebacterium glutamicum* (CGMCC1.1886), *Micrococcus lysodeikticus* (CGMCC1.634), *Bacillus subtilis* (CGMCC1.3358), *Escherichia coli* (CGMCC1.2389), *Pseudomonas aeruginosa* (CGMCC1.2421), *Pseudomonas fluorescens* (CGMCC1.3202), *Pseudomonas stutzeri* (CGMCC1.1803)*, Aeromonas* *hydrophila* (CGMCC1.2017), *Shigella flexneri* (CGMCC1.1868) were purchased from CGMCC (China General Microbiological Culture Collection Center, Beijing, China).

The minimum inhibitory concentration (MIC) of rSpCrus8 and rTrx-SpCrus8 were determined in triplicate on separated occasions following growth inhibition assay protocol described previously (Bulet et al., 1993). Briefly, rSpCrus8 and rTrx-SpCrus8 were serially diluted with Milli-Q water and kept on ice until use. Bacteria were harvested in the logarithmic growth phase, washed with 10 mM Dulbecco’s phosphate buffered saline (DPBS, pH 7.4) and adjusted to 3.3 × 10^4^ CFU mL^−1^ prior to incubation with rSpCrus8 or rTrx-SpCrus8. After 24 h of incubation, MICs were then determined. All experiments were repeated three times.

**Table S1 Primers used in the study.**

| **Primers** | **Sequence（5′-3′）** |  |
| --- | --- | --- |
| **cDNA cloning** |  |  |
| SpCrus8-CDS-F | ATGAAGACGACGGTGCTACTGC |  |
| SpCrus8-CDS-R | TCAGCCGAACTGTATCGGTGG |  |
| SpCrus8-3′-F1 | CCAAAGCCAAGGAACCTCGC |  |
| SpCrus8-3′-F2 | CGCATTTGCTTTGCTTCCATC |  |
| SpCrus8-5′-R1 | GTCTGTCCCAACACTCGCAG |  |
| SpCrus8-5′-R2 | CAACCTGCTCCAATGATGGC |  |
| **qPCR** |  |  |
| SpCrus8-qPCR-F | ATGAAGTGGGATTCGGCGGC |  |
| SpCrus8-qPCR-R | ATGTCCTCCCAAAACGGGGC |  |
| SpRab5-qPCR-F | TGGAGACGTCCGCCAAAACT |  |
| SpRab5-qPCR-R | GGGTCATCCCTTCCCGTTGG |  |
| SpRab7-qPCR-F | CAGACTGCTGTGTGCTCTGCT |  |
| SpRab7-qPCR-R | TGGTCTGGGTCACGAGGTGAT |  |
| SpIMD-qPCR-F | GGTCGTCAACATCACGGGCA |  |
| SpIMD-qPCR-R | CTCTCGCATCTGAGCAGGGC |  |
| SpTAK1-qPCR-F | GGCAGTGAAGAAGGTGGAGACAGA |  |
| SpTAK1-qPCR-R | CAGCCTTGGCACACTGGAACA |  |
| SpIKKβ-qPCR-F | CACGGCTTCTGGCTCTCCTGAT |  |
| SpIKKβ-qPCR-R | GATGGCGGGCTTGTAACTTTGCTA |  |
| SpIKKε-qPCR-F | GGTCATGCAGTCAAGCGCAAGA |  |
| SpIKKε-qPCR-R | TGCACGTGGTTCAGCTGTGA |  |
| SpRelish-qPCR-F | AGTGGAACAGTGGTCCAGCTG |  |
| SpRelish-qPCR-R | CACCACCACTTCACAAATC |  |
| SpCrus3-PCR-F | ACCTGCCTGGCCATTACGTG |  |
| SpCrus3-PCR-R | CCCACCACAGGGAGTGTTGC |  |
| SpCrus4-PCR-F | CCTGCCTGGCCATTACGTGT |  |
| SpCrus4-PCR-R | GCTTGCACACCTTCGCTTCG |  |
| SpCrus5-qPCR-F | TAGCGTTCCTCGTGCTGGTG |  |
| SpCrus5-qPCR-R | ACGAATTGGAGGGCAGCGTC |  |
| SpGAPDH-F | ACCCATGTTTGTGTGTGGTG |  |
| SpGAPDH-R | ACAGTGGTCATGAGGCCCTG |  |
| **Protein expression** | |  |
| ExSpCrus8-F | CGC**CATATG**GAAAAGAAAGAAGGGAAGTC |  |
| ExSpCrus8-R | CCG**CTCGAG**TCA**ATGGTGATGGTGATGATG**GCCGAACTGTATCGGTG |  |
| ExTrx-SpCrus8-F | CCG**GAATTC**GAAAAGAAAGAAGGGAAGTC |  |
| ExTrx-SpCrus8-F | CCC**CTCGAG**TCAGCCGAACTGTATCGGTG |  |
| **dsRNA synthesis** |  |  |
| dsSpCrus8-F | TAATACGACTCACTATAGGNGACACCACGGGATCAGTTCT |  |
| dsSpCrus8-R | TAATACGACTCACTATAGGNCGAAGCAGCACTTGTCGTAG |  |
| dsGFP-F | TAATACGACTCACTATAGGGAGATGAGCAAGGGCGAGGAGCTGTTCAC |  |
| dsGFP-R | TAATACGACTCACTATAGGGAGATTACTTGTACAGCTCGTCCATGCCG |  |
| **Primers** | **Sequence（5′-3′）** |  |
| **RNAi efficiency detection** | |  |
| dtSpCrus8-F | GCAAATGCGAGGTTCCTTGG |  |
| dtSpCrus8-R | GAGTGTTGGGACAGACGGG |  |

**Table S2 Amino acid sequence identities of crustin genes with SpCrus8 in *S. paramamosain*.**

| **Gene name** | **Type** | **GenBank accession NO.** | **Amino acid sequence identities** | |
| --- | --- | --- | --- | --- |
|  |  |  | **CDS** | **WAP domain** |
| CrusSp | Type I | EU161287 | 38.96% | 46.81% |
| SpCrus3 | Type I | MF431587 | 45.00% | 45.00% |
| SpCrus4 | Type I | MF431588 | 44.44% | 40.62% |
| SpCrus6 | Type I | MF431585 | 37.93% | 44.90% |
| SpCrus7 | Type I | MK733605 | 38.82% | 51.16% |
| SpCrus2 | Type II | MF431589 | 41.03% | 52.17% |
| SpCrus5 | Type II | MF431586 | 55.56% | 63.27% |
| SpCrus8 | Type II | MZ826792 | - | - |

**Table S3 The antibacterial activity of rSpCrus8 and rTrx-SpCrus8.**

| **Microorganism** | **CGMCC NO.** | **MIC (μM)** | |
| --- | --- | --- | --- |
|  |  | rTrx-SpCrus8 | rSpCrus8 |
| **Gram-positive bacteria** |  |  |  |
| *Staphylococcus aureus* | 1.2465 | >48 | >48 |
| *Listeria onocytogenes* | 1.10753 | >48 | >48 |
| *Enterococcus faecalis* | 1.2135 | >48 | >48 |
| *Corynebacterium glutamicum* | 1.1886 | >48 | >48 |
| *Staphylococcus epidermidis* | 1.4260 | >48 | >48 |
| *Micrococcus lysodeikticus* | 1.634 | >48 | >48 |
| *Bacillus subtilis* | 1.3358 | >48 | >48 |
| **Gram-negative bacteria** |  |  |  |
| *Escherichia coli* | 1.2389 | >48 | >48 |
| *Pseudomonas aeruginosa* | 1.2421 | >48 | >48 |
| *Pseudomonas fluorescens* | 1.3202 | >48 | >48 |
| *Pseudomonas stutzeri* | 1.1803 | >48 | >48 |
| *Aeromonas* *hydrophila* | 1.2017 | >48 | >48 |
| *Shigella flexneri* | 1.1868 | >48 | >48 |


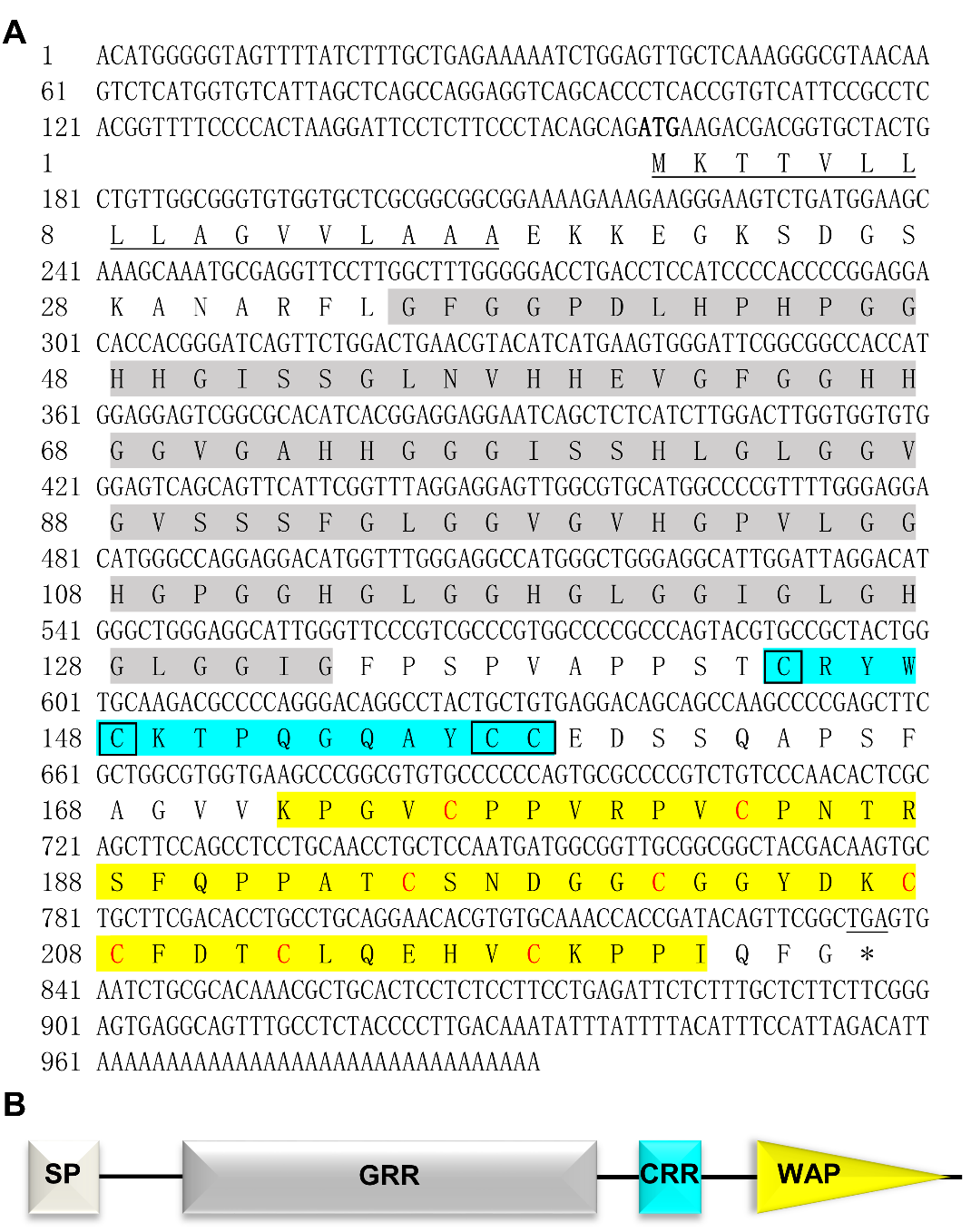


**Fig. S1 Amino acid sequence and bioinformatics analysis of SpCrus8.** (A). Nucleotide sequence and deduced amino sequence of SpCrus8 from *S. paramamosain*. Start codon (ATG) is marked in bold and the stop codon (TGA) is underlined with “*”. The signal peptide is underlined, the GRR is shaded in gray, the CRR is shaded in blue and the WAPD is shaded in yellow. Cysteine residues in the CRR and the WAPD are marked with boxes and red, respectively. (B). Schematic diagram of the structure of the SpCrus8 protein predicted by the SMART database.

**
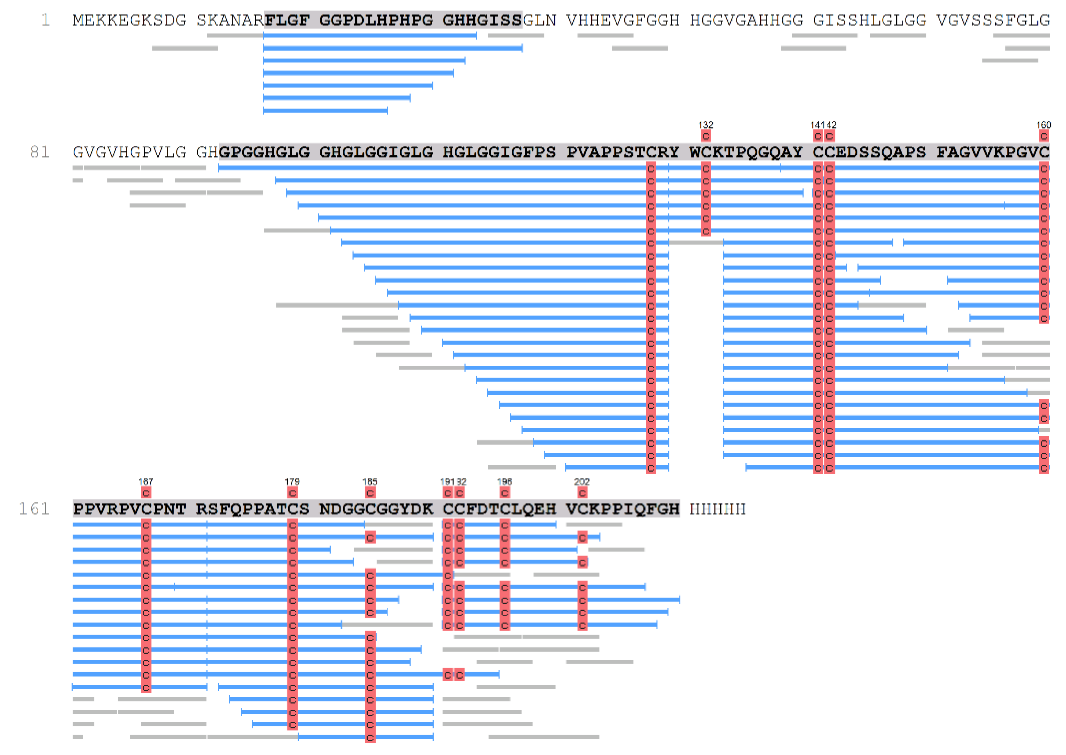
Fig. S2 Mass spectrometry analysis of rSpCrus8.**

**

**

**Fig. S3 The binding activity of SpCrus8 recombinant proteins to PAMPs.**

LTA: lipoteichoic acid, LPS: lipopolysaccharide, PGN: peptidoglycan, GLU: glucan. The protein concentration for rTrx, rSpCrus8 and rTrx-SpCrus8 was 1 μM. Significant difference was indicated with asterisks, ***p < 0.001.

**
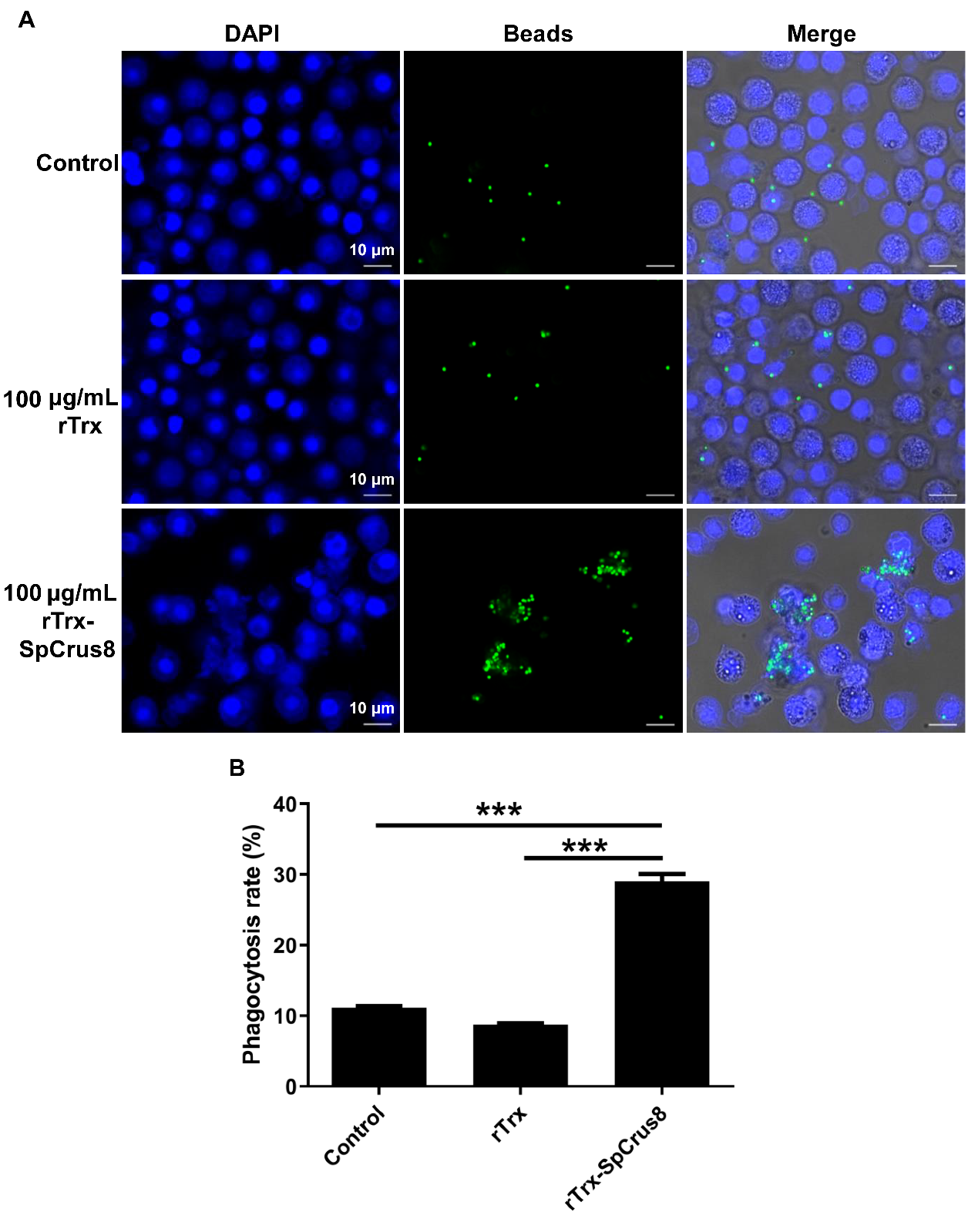
Fig. S4 Phagocytosis of fluorescent microspheres by hemocytes after rTrx-rSpCrus8 treatment.** (A). Phagocytosis of fluorescent microspheres by hemocytes after rTrx-SpCrus8 or rTrx treatment (100 μg/mL) was observed under CLSM, and equal volumn of L-15 medium was used as control. The nuclei of hemocytes were stained with DAPI (blue), and green fluorescence represented fluorescent microspheres. (B). The phagocytosis rate of fluorescent microspheres by hemocytes was determined by flow cytometry. Significant difference was indicated with asterisks, ***p<0.001.

**Reference**

Bulet, P., Dimarcq, J.L., Hetru, C., et al., 1993. A novel inducible antibacterial peptide of Drosophila carries an O-glycosylated substitution. J Biol Chem 268, 14893-14897.
